# Canopy Structure, Light Intensity, Temperature and Photosynthetic Performance of Winter Wheat under Different Irrigation Conditions

**DOI:** 10.3390/plants12193482

**Published:** 2023-10-05

**Authors:** Meng Zhang, Weiwei Chen, Maoya Jing, Yanmei Gao, Zhimin Wang

**Affiliations:** 1 School of Life Science, Shanxi Normal University, Taiyuan 030031, China; sprout205@163.com (M.Z.);; 2 College of Agronomy and Biotechnology, China Agricultural University, Beijing 100193, China; zhimin206@263.net

**Keywords:** wheat, canopy architecture, non-leaf organs, canopy apparent photosynthesis

## Abstract

A high-quality canopy architecture is central to obtaining high crop yields. A field experiment was carried out at the Wuqiao Experimental Station from 2015 to 2019 under four irrigation schemes (W_0_, no irrigation after sowing; W_1_, 75 mm irrigation at jointing stage; W_2_, 75 mm irrigation at jointing and anthesis stages, respectively; W_3_, 75 mm irrigation at tillering, jointing and anthesis stages, respectively) to investigate the canopy structure, canopy apparent photosynthesis (CAP), canopy temperature (CT), yield and water use efficiency (WUE). The results showed that increasing irrigation times improved the leaf area index (LAI), non-leaf area index (NLAI) and light interception (LI) of the spike and total canopy but decreased the canopy temperature (CT) after anthesis. The CAP in the W_3_ treatment was consistently lower than that in the W_1_ treatment, suggesting lower effective utilization of light energy under the W_3_ treatment. Increasing irrigation times improved wheat yield, but the W_2_ treatment had no significant difference in yield compared to the W_3_ treatment. In addition, the W_1_ and W_2_ treatments had higher WUEs. The CT, organ temperature and LI were closely positively associated with each other, but they were all strongly negatively related to the yield. Overall, the W_2_ treatment was the best irrigation scheme for constructing a reasonable canopy architecture for winter wheat, obtaining more efficient water use and yield in the North China Plain (NCP). CT and organ temperature can be used as proxy parameters to estimate the canopy structure.

## 1. Introduction

Winter wheat (*Triticum aestivum* L.) is the most widely planted and important global grain crop [1]. Improving wheat production is imperative for ensuring an adequate supply of food for the expanding global population [2]. A favorable crop population architecture is the foundation for obtaining high crop yields and water productivity. Crop population architecture will affect canopy light distribution and light interception, which is determined by many factors, including genotype, water, density, nitrogen and their interactions [3,4,5]. However, increases in frequent and irregular water stress have become the main limiting factor for wheat productivity in the North China Plain (NCP). In this region, the average water consumption in winter wheat growing season is approximately 450 mm, which is two to three times the average rainfall [6]. Irrigation is required to guarantee the water demand for winter wheat production [7,8]. But, overexploitation of groundwater has caused the rapid decline of groundwater levels, inducing serious environmental problems such as land subsidence and threats to sustainable agricultural development [9,10]. Thus, developing a high-quality population structure with efficient light energy utilization and WUE under water-saving conditions is important for thoroughly resolving water scarcity. 

Droughts alter plant morphological (e.g., declines in leaf area, length, and width) and physiological traits (e.g., decrease in leaf chlorophyll concentrations and maximum photosynthesis) [11]. Irrigation affects leaf growth and expansion, which is positively correlated with water supply [12,13]. Controlling soil water in the spring can control leaf size and reduce unnecessary water consumption [14]. Xu [15] indicated that irrigation at the jointing stage leads to reduced areas in the upper three leaf layers at anthesis compared to the first irrigation at the late tillering stage. He [16] also reported that the leaf area gradually increased with decreasing delays in irrigation, and that irrigation at a particular stage when spring leaves emerged could significantly increase the areas of the next and subsequent leaves. Irrigation also affects green non-leaf organ areas (e.g., spike, peduncle and sheath). Zhang [17] showed that an increase in the irrigation amount improved the spike area per unit of soil area. A reduced water supply decreased the wheat photosynthetic organs’ area, and the leaf area decreased much more quickly than the non-leaf area [12]. The ratio of green non-leaf organs to the total green area increased with reduced irrigation amounts [18].

Irrigation influences tillers and spike numbers. After irrigation, extractable soil water is mainly stored in the 0–60 cm soil layers, where soil water content has been positively correlated with tiller number [19]. Increasing the irrigation amount is beneficial for improving the effective tiller [20]. Zhao [21] reported that moderate irrigation at the jointing stage increased the spike number. Optimizing soil moisture during jointing increased sugar metabolism in low-position tillers and converted them into effective spikes [22,23].

Canopy architecture affects light interception and the efficient utilization of light energy [24]. The canopy structure of wheat could be shaped by a controlled irrigation regime. He [16] found that the light interception above the flag leaf gradually reduced, but the light interception for the penultimate leaf, the remaining leaves, and the CAP increased under reduced irrigation conditions. Gao [12] proposed that a greater water supply could improve the light interception ratio of flag leaves but, it also decreased the light interception ratio of the lower layers of leaves. Reducing the leaf area of the upper layers could improve the light exposure in the lower layers. However, a reduction in the leaf water potential of the plant caused by a reduction of soil moisture ultimately reduces photosynthesis [25].

Canopy architecture attributes such as the angle of leaves and the degree of shading in the canopy also influence microclimate conditions [26]. Studies have shown that canopy temperature (CT) is associated with yield, water use efficiency (WUE), leaf area index (LAI) and the stomatal conductance of winter wheat [26,27,28]. However, not all studies have shown a correlation between CT and yield [29]. Studying the CT and its mechanism is helpful for accurately understanding the microenvironment of wheat and establishing relationships with crop yield, as well as providing support for water management decisions. Screening for CT has been used in field phenotyping experiments for wheat [30] and has been successfully integrated into practical breeding programs [31]. 

The normalized difference vegetation index (NDVI) is one of the best-known multispectral indices that helps to evaluate plant biomass, LAI and net primary productivity [32]. Tan [33] integrated the NDVI and Beer–Lambert law to estimate the dynamic changes of wheat canopy architecture parameters. Thapa [34] found that anthesis to the middle of the grain filling period was the most suitable time to characterize winter wheat’s response to water stress based on the NDVI.

To date, studies have been conducted on the interaction effect of irrigation on wheat canopy characteristics and yield formation, with a primary focus on the canopy architecture’s leaf size, leaf angle and light interception. However, there have been fewer studies exploring the non-leaf green organ characteristics, microclimate and diurnal changes of the CAP. We hypothesized that appropriate cultivation practices could build a reasonable canopy structure and create a suitable light and temperature microenvironment, which improves population photosynthesis and ultimately improves crop yield and water use efficiency. Therefore, the objectives of this study were: (1) to compare the area and ratio of the photosynthetic organs in single plants; (2) to study the light interception for different organs; (3) to determine how the canopy structure parameters were correlated with yield; and (4) to test the use of CT and NDVI as a proxy to estimate canopy architecture.

## 2. Results

### 2.1. Soil Water Content

**Figure 1:**
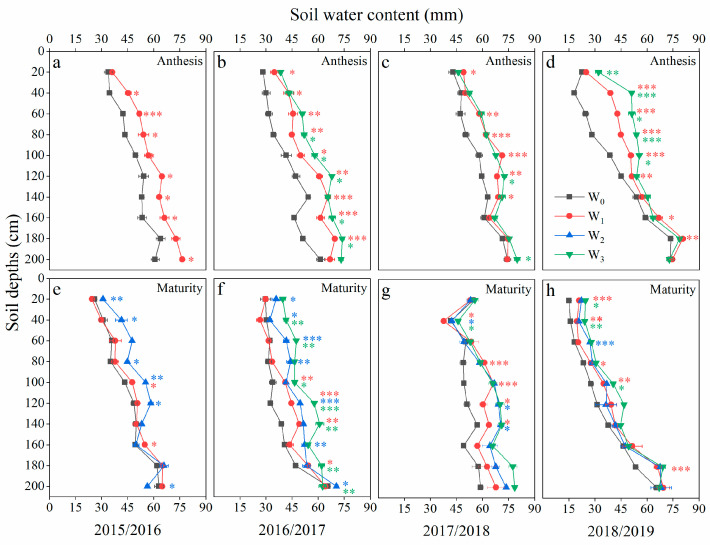
Soil water content at a depth of 0–200 cm with different irrigation treatments at anthesis (**a**–**d**) and maturity (**e**–**h**). The red asterisks indicate significant differences between W_0_ and W_1_, the green asterisks indicate significant differences between W_3_ and W_2_, and the blue asterisks indicate significant differences between W_2_ and W_1_.

Irrigation is one of the key factors affecting soil water. Compared with the W_0_ treatment, the W_1_ treatment significantly improved the soil water content (SWC) of the 0–180 cm soil layer at the anthesis stage in 2015/2016, 2016/2017 and 2018/2019. It also significantly improved the SWC of the 0–140 cm soil layer in 2017/2018, and it had no effect at the deeper layers (Figure 1). Compared with the W_1_ treatment, the W_3_ treatment significantly increased the SWC in the 80–180 cm soil layer in 2016/2017 and improved it within a 0–100 cm soil depth in 2018/2019 at the anthesis stage, while the W_3_ and W_1_ treatments exhibited no significant differences in 2017/2018. At maturity, compared with the W_0_ treatment, the W_1_ treatment improved the SWC of the 80–140 cm soil layer. The W_2_ treatment significantly increased the SWC in the 0–120 cm soil layer in 2015/2016 and 2016/2017 and had no effect on it in 2017/2018 or 2018/2019 when compared with the W_1_ treatment. The W_3_ treatment had a higher SWC in the 0–200 cm than the W_2_ treatment in 2016/2017.

### 2.2. Canopy Architecture

#### 2.2.1. The Area and Ratio of the Photosynthetic Organs

The leaf blade had the maximum area and ratio at anthesis as the main photosynthetic organ, followed by the stem and spike (Table 1). The leaf area and spike area increased with increasing irrigation times, and the area of the leaf increased much more quickly than the stem and spike areas. Compared with W_0_, the W_1_ and W_3_ treatments increased the leaf area by averages of 19.3% and 59.6%, respectively, and increased the spike area by averages of 7.4% and 19.8%, respectively. The impact of irrigation on the total green organ area showed a very similar trend to the effects of irrigation on leaf area. The leaf ratios were greater under the W_1_ and W_3_ treatments, while the ratios of non-leaf organs (stem and spike) were higher under the W_0_ treatment. The trends in irrigation effects on the total green organ area were consistent with the leaf area.

#### 2.2.2. Canopy Green Organs’ Area Index

At anthesis, the NLAI approached or even exceeded the LAI, especially in the W_0_ treatment (Table 2). The GAI, LAI and NLAI increased with water input, while the NLAIs under the W_1_ and W_3_ treatments were similar, and both were significantly higher than those under the W_0_ treatment.

#### 2.2.3. Light Interception

**Figure 2:**
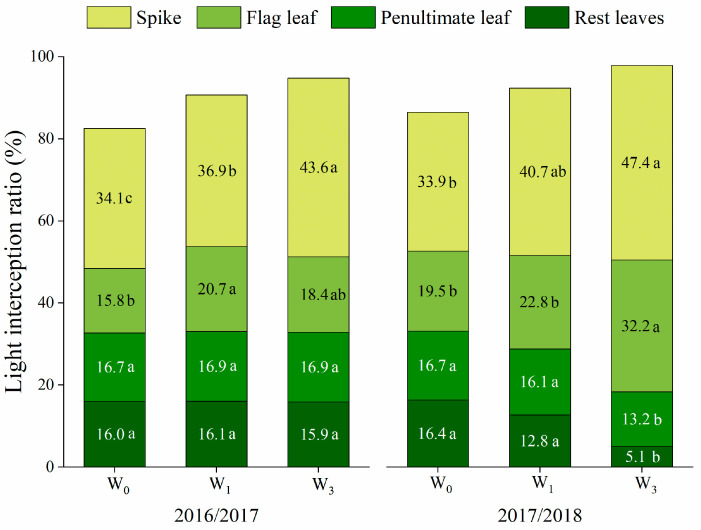
Light interception ratio of spike, flag leaf, penultimate leaf and the rest of the leaves under different irrigation treatments in 2016/2017 and 2017/2018. Different lowercase letters indicate significant differences based on an LSD test (*p* < 0.05) among the irrigation treatments.

Irrigation increases the total light interception ratio of the wheat canopy, which ranged from 86.5% to 97.9% (Figure 2). The spikes intercepted the most light due to their topside advantage, and the light interception ratio increased with an increasing water supply. Irrigation increased the light interception ratio of the flag leaf, especially under the W_1_ treatment in 2016/2017 and the W_3_ treatment in 2017/2018. In 2016/2017, increasing the irrigation times had no effect on the light interception ratio of the penultimate leaf and the rest of the leaves, but there was a significant decrease under the W_3_ treatment during the 2017/2018 season.

### 2.3. Temperature of Canopy and Organs

#### 2.3.1. Canopy Temperature and Canopy Temperature Depression

The CT and CTD values under different irrigation treatments after anthesis are presented in Table 3. The CT after anthesis decreased significantly with increasing irrigation times. In the two growth seasons, the CT was generally lower than the air temperature, resulting in positive CTD values. The change in trend of the CTD was opposite to that of the CT; the CTD after anthesis increased under the irrigation treatment. In 2016/2017, there were significant differences in the CTD between the W_1_ and W_2_ treatments, but in 2017/2018, there were no significant differences.

#### 2.3.2. Temperature of Organs

The temperature of the spike was significantly higher than the temperature of the other organs at anthesis and DAA10 (Table 4). Irrigation tended to significantly decrease the temperature of all the wheat organs. Among all the wheat organs, the spike experienced the largest decrease due to irrigation, ranging from 0.92 to 2.59 °C.

### 2.4. Canopy Photosynthetic Rate and Respiration Rate

#### 2.4.1. Canopy Apparent Photosynthesis at Different Growth Stages

**Figure 3:**
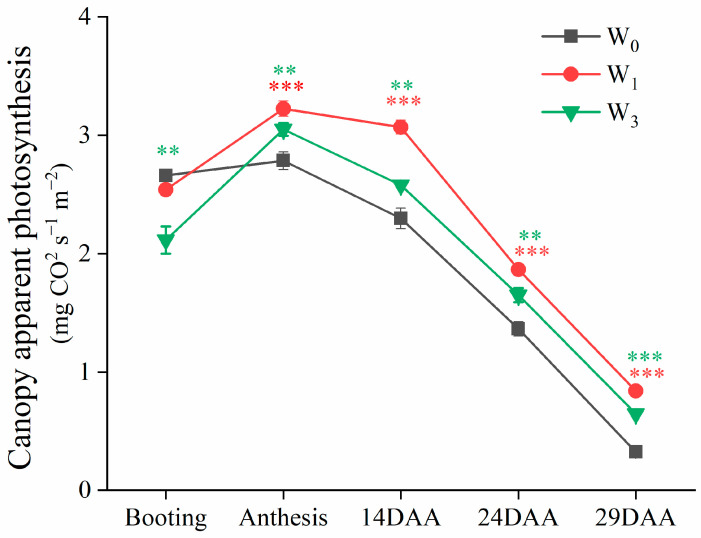
Canopy apparent photosynthesis (CAP) under different irrigation treatments at different growth stages in 2017/2018. Vertical bars represent the mean SE of three replications per treatment. The red asterisks indicate significant differences between W_0_ and W_1_, and the green asterisks indicate significant differences between W_3_ and W_0_.

The variation trends in the CAP were similar across the different irrigation treatments (Figure 3). The CAP values increased during the booting stage, reached a peak at the anthesis stage, and then decreased during the grain filling stage. The CAP under the W_3_ treatment at the booting stage was significantly lower than that in the W_0_ and W_1_ treatments. At anthesis and the grain filling stage, the CAP under the W_1_ treatment was significantly higher than in the other treatments. Compared to the CAP at anthesis, the CAPs at 14 DAA in the W_1_, W_3_ and W_0_ treatments were decreased by 4.85%, 15.41% and 17.49%, respectively.

#### 2.4.2. Diurnal Variation of Canopy Apparent Photosynthesis and Respiration Rate

**Figure 4:**
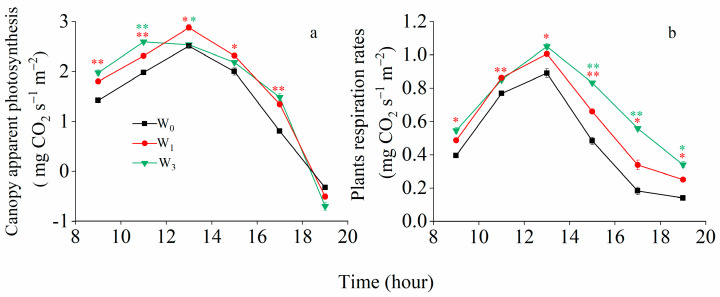
Diurnal variations in canopy apparent photosynthesis (**a**) and respiration rates (**b**) under different irrigation treatments at 1 DAA in 2017/2018. Vertical bars represent the mean SE of three replications per treatment. The red asterisks indicate significant differences between W_0_ and W_1_, and the green asterisks indicate significant differences between W_3_ and W_1_.

The diurnal variation of the CAP rate and respiration rates were measured with an average temperature of 16.7 ℃ and a maximum temperature 23.5 °C at 1 DAA (Figure 4). The CAP increased to the maximum close to noon and decreased in the afternoon. The maximum value appeared no later than 14:00. The diurnal variations in CAP under the W_0_ and W_1_ treatments reached their maximum from 12:00 to 14:00, and the diurnal variations in CAP under the W_3_ treatment peaked between 10:00 and 12:00. The diurnal variations in canopy respiration rates followed a similar trend to the CAP. The maximal respiration rates were 0.89, 1.05 and 1.01 mg CO_2_ s^−1^ m^−2^ under the W_0_, W_1_ and W_3_ treatments, respectively. Compared to the W_0_ treatment, the irrigation significantly increased the CAP from 8:00 to 18:00 and significantly increased the respiration rates from 8:00 to 20:00.

### 2.5. Normalized Difference Vegetation Index

**Figure 5:**
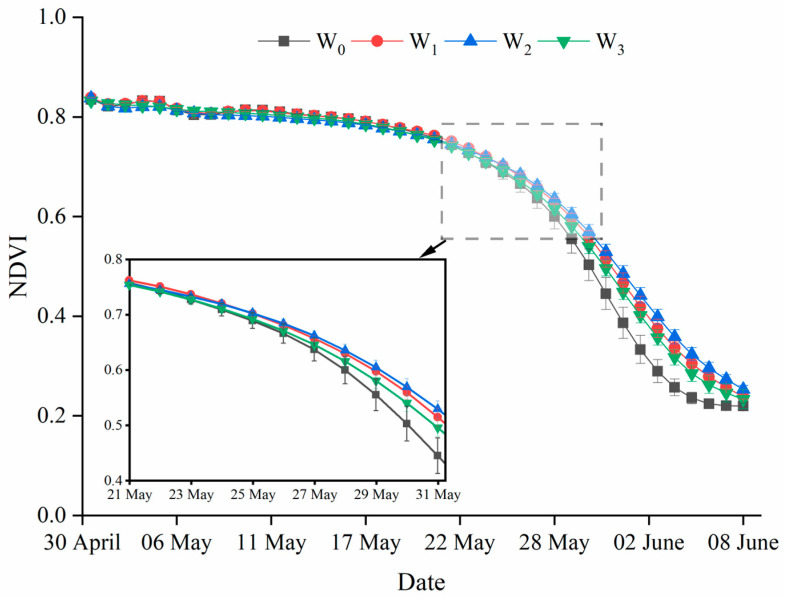
NDVI values under different irrigation treatments in 2017/2018. Vertical bars represent the mean SE of the thirty replications per treatment.

Figure 5 displays the time courses of NDVI for different irrigation treatments from the booting stage to maturity. The NDVI values demonstrated that W_2_ > W_1_ > W_3_ > W_0_, and in general, the NDVI values decreased gradually from booting to maturity. The NDVI values were higher for the W_2_ treatment than the other treatments during the middle to later grain filling stage. The NDVI decreased at a slower rate from anthesis to mid-grain filling under the W_0_ treatment, but with a more rapid decline and lower NDVI values in the late season.

### 2.6. Yield, ET and WUE

Generally, the yield and ET increased with increasing irrigation amounts (Table 5). The wheat under W_3_ achieved the highest yield (averaged 8077 kg ha^−1^) and ET (averaged 474.0 mm), but there was no significant difference in grain yield between the W_3_ and W_2_ treatments except for the yield in 2018/2019. The WUE of winter wheat showed an increasing and then decreasing trend with increasing irrigation times, and the W_1_ and W_2_ treatments had the highest WUE.

### 2.7. Correlation Analysis

**Figure 6:**
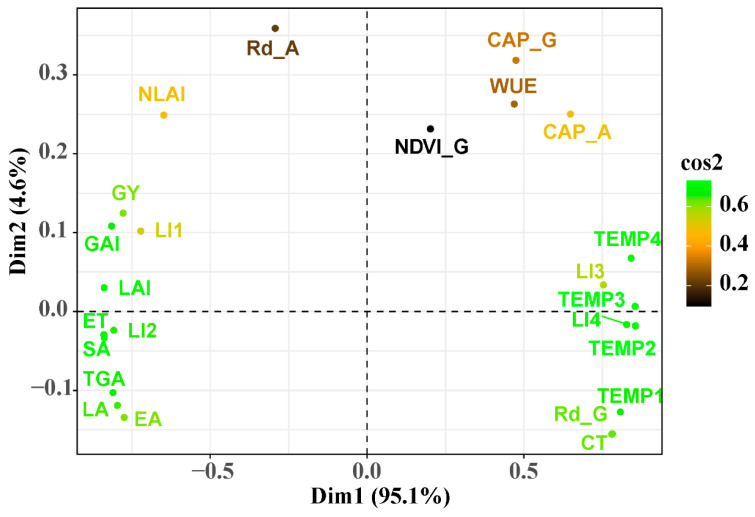
A principal component analysis (PCA) was conducted based on the date of yield, proxy indicators, canopy photosynthetic parameters, and canopy architecture in 2017/2018. Note: NDVI at the grain filling stage (NDVI-G), leaf area (LA), stem area (SA), spike area (EA), total green area (TGA), leaf area index (LAI), non-leaf area index (NLAI), total green area index (GAI), canopy apparent photosynthesis at anthesis (CAP-A), canopy apparent photosynthesis at 14DAA (CAP-G), plant respiration rates at anthesis (Rd-A), plant respiration rates at 14DAA (Rd-G), crop evapotranspiration (ET), yield (GY), water use efficiency (WUE), light interception ratio of spike (LI1), light interception ratio of the flag leaf (LI2), light interception ratio of the penultimate leaf (LI3), light interception ratio of the rest of the leaves (LI4), canopy temperature (CT), temperature of spike (TEMP1), temperature of the flag leaf (TEMP2), temperature in the middle of the stem (TEMP3), temperature at the base of the stem (TEMP4).

A principal component analysis (PCA) was conducted to explain the relationship between proxy indicators (i.e., CT and NDVI), canopy photosynthetic parameters and canopy architecture and yield (Figure 6). The yield was very significantly positively correlated with the GAI, LAI and LI of spike at anthesis. The organ temperatures and light intercept of the lower layers were strongly negatively related to the yield. The organ green area and ET were closely positively related, but negatively related with the WUE. Nevertheless, there was no clear correlation between NDVI and the other indicators.

## 3. Discussion

Irrigation is the most widely used way to combat soil water deficiency and improve winter wheat yields in semi-arid and arid regions [6]. However, poor irrigation management results in unreasonable canopy structures, leading to disruptions in WUE [35]. Optimal irrigation scheduling can promote root growth into deeper layers of the soil and water absorption from a wide soil rhizosphere, relatively optimizing wheat canopies and regulating water consumption [36]. In this study, the canopy architecture and microenvironment were affected by the irrigation treatment. As the irrigation amount increased, the leaf area and spike area increased, while the proportion of non-leaf area to total green area per stem decreased (Table 2). In this study, we did not measure the area of the awn directly; instead the spike surface area was calculated as a cylinder using a compromise method. The spike surface area measured using this method was close to the observed spike surface area (with awns). This method may be applicable only to wheat varieties with the same spike type as this study. The non-leaf area comprised approximately half of the total green area per stem. However, the results of our study were inconsistent with the findings of Zhang [18], who reported that the ratio of the non-leaf area to the total green area of wheat was 63%. This is due to the different methods of determining the area of non-leaf organs. The previous method measured the total surface area of non-leaf organs rather than the effective surface area for light interception, and this may overestimate the non-leaf organs’ areas. In spite of this variability, the results obtained using different methods support the importance of the non-leaf organs as major photosynthesis organs.

The green area index is an important index of canopy architecture. In this study, increasing irrigation times improved the LAI and NLAI at anthesis in the successive 4 years (Table 2). Thus, the amount of light intercepted by the canopy was increased, especially by the spike in the upper strata (Figure 2). The improvement in the photosynthetic rate that was observed with increasing irrigation was mainly due to the change in the canopy architecture, the increase in the GAI and canopy light interception, and the decrease in light leakage loss [37,38]. Previous studies have indicated that drought and weak incident light accelerates leaf senescence [12,39]. This phenomenon was found in the current study, which was reflected in the NDVI, as shown by the lower values under the W_0_ and W_3_ treatments during post-anthesis (Figure 5). The wheat plants’ NDVI declined rapidly due to increased drought conditions under the W_0_ treatment, while a deterioration in the light environment in the lower layers led to faster NDVI declines under the W_3_ treatment. Although NDVI is a quick and easy way to measure the stay-green and plant status, it cannot reflect vertical changes in the canopy structure [32].

Photosynthesis is the primary determinant of crop yield, and CAP is closely related to the canopy architecture and positively correlated with light interception [40]. The wheat canopy is complex and heterogeneous, and it was difficult to accurately measure the amount of light intercepted by each organ. In this study, we divided the wheat canopy into different layers from top to bottom, and depending on the dominant organ in the different layers, we estimated the light interception of the different organs. The CAP accurately describes the photosynthetic capacity per unit area of a crop and integrates the characteristics of the canopy structure [41]. In this study, the CAP under the W_3_ treatment was consistently lower than under the W_1_ treatments during the post-anthesis period (Figure 3), while there was no significant difference in the SWC at 0–200 cm between the W_1_ and W_3_ treatments at the anthesis stage (Figure 1c,g). This is because the light microclimate deteriorated quickly from the top to the bottom of the canopy and was aggravated with an increasing water supply. The light redundancy in the upper layers resulted in a poorer light environment in the lower layers, in which respiration exceeded photosynthesis (below the light compensation point), causing light starvation in the lower layers. The differences in CAP between the W_3_ and W_1_ treatments were due to irrigation-induced changes in the canopy structure and not the soil moisture. The diurnal photosynthesis amount was highest in the W_1_ treatment, followed by W_3_ treatment, and were lowest in the W_0_ irrigation treatment. The diurnal respiration amounts were 2.9, 3.6 and 4.2 mg CO_2_ s^−1^ m^−2^ under the W_0_, W_1_ and W_3_ treatments, respectively. The W_1_ treatment reduced the leaf area relative to the W_3_ treatment, improved the canopy architecture, reduced canopy respiration and maintained a higher daily CAP, and its yield and WUE were also higher.

Canopy structure can affect canopy temperature, and canopy temperature differs between irrigation treatments [42]. In this study, the CT after anthesis decreased significantly with increasing irrigation times (Table 3). Previous studies have reported that temperature differences of up to 5 °C were observed among the spikes between irrigated and rainfed crops on a hot day [43]. In our study, irrigation tended to significantly decrease the temperature of all wheat organs, and the spike experienced the largest decrease due to irrigation, ranging from 0.92 to 2.59 °C (Table 4). The position of the spikes ensures a minimum amount of shading, and the transpiration of the spike is lower than that of the flag leaves. Therefore, the spike temperature was higher than the leaf temperature and slightly lower than the CT. In our study, the spikes had the highest temperature among the plant organs at anthesis and the early grain filling stage. This study found significant correlations between CT and yield, and the organ temperature of central and base of the stem were closely positively related with the light intercept of lower layers. The CT and organ temperature can be important parameters reflecting the ecological and physiological state of the plant [44].

The correlations among the canopy architecture, microclimate and yield disclosed that the GAI, LAI and LI of the spike were negatively correlated with the yield. The spike played an important role in grain yield, and its canopy characteristics can be proxy measures of grain yield [17]. Increasing the number of irrigations increased the yield, but there was no significant difference in yield between the W_2_ and W_3_ treatments (Table 5). The effect of irrigation on winter wheat yield and WUE have been well studied. Appropriate increases in irrigation can improve the yield, but excessive irrigation will reduce the wheat yield and WUE [45]. In our study, the WUE first increased and then decreased as the amount of irrigation time increased from 2016 to 2018, and the WUE was highest under the W_2_ treatment (1.80 and 1.95 kg m^−3^, respectively). However, the WUE was highest under the W_1_ treatment in 2018/2019 due to a deficiency in precipitation (56.2 mm) during the growing period of the winter wheat. However, this study only explored the effects of irrigation on canopy structure and light intensity and did not accurately measure the photosynthetic areas of non-leaf organs and light interception of different organs. Further research is needed to more accurately characterize canopy structure and examine the interactions between soil nutrients (e.g., nitrogen) and varieties.

## 4. Materials and Methods

### 4.1. Experimental Site and Experimental Design

**Figure 7:**
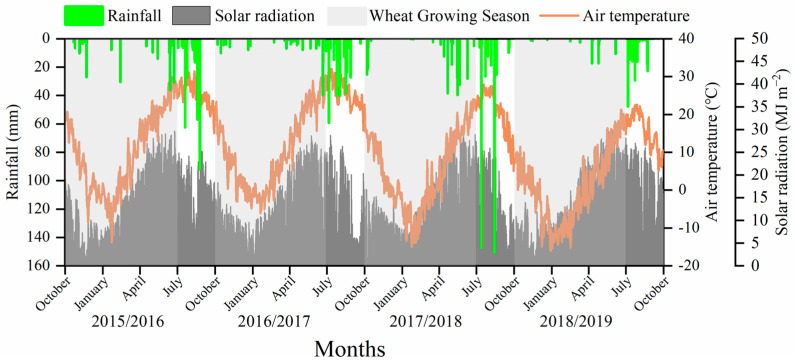
The daily rainfall, solar radiation and mean air temperature from 2015 to 2019 at the Wuqiao Experimental Station.

The field experiments were carried out in 2015/2016, 2016/2017, 2017/2018 and 2018/2019 at the experimental station at the China Agricultural University, China, located in Wuqiao, Hebei Province. The climate is summer monsoon climate with an average annual precipitation of 562 mm, but with only 30% precipitation concentrated on the winter wheat growth season (October to July). The solar radiation, air temperature and rainfall from 2015 to 2019 are shown in Figure 7. The precipitation received during the winter wheat seasons from 2015 to 2019 was 153.2, 87.8, 191.5 and 56.2 mm, respectively. The soil was clay–loam with 1.25% organic matter, 1.02 g kg^−1^ total nitrogen (N), 31.6 mg kg^−1^ available phosphorus (P) and 48.1 mg kg^−1^ available potassium (K).

The experiments were based on a randomized block design with three replications. The plot area was 40 m^2^ (5 m × 8 m). ‘Nongda399’, a modern awned semi-dwarfing winter wheat variety was sown on 11 October 2015, 12 October 2016, 21 October 2017, and 15 October 2018 with a row space of 20 cm. The seeding densities were 600 plants m^−2^ from 2015 to 2017, and 750 plants m^−2^ in 2018 due to delayed sowing. All treatments received a pre-sowing irrigation of 100 mm in both years of study (Table 6). In 2015/2016, three irrigation treatments were designed, i.e., W_0_., no irrigation during the growing stage; W_1_, 75 mm of total irrigation with 75 mm irrigated at the jointing stage; and W_2_, 150 mm of total irrigation with 75 mm irrigated at the jointing and anthesis stages, respectively. In 2016/2017, 2017/2018 and 2018/2019 an additional irrigation treatment was established: W_3_, 255 mm of total irrigation with 75 mm irrigated at the tillering, jointing and anthesis stages, respectively. W_3_ is the conventional irrigation for winter wheat and is used by local farmers, W_2_ is currently promoted by the local government and allows for a high yield and water use efficiency (WUE) and W_1_ and W_0_ are irrigations with a more reduced frequency to obtain basic data to further conserve irrigation water. All experiments received 225 kg N ha^−1^ (as urea), 300 kg P ha^−1^ (as superphosphate) and 225 kg K ha^−1^ (as potassium sulfate) before sowing. Pests and diseases were properly controlled.

### 4.2. Measurements

#### 4.2.1. Green Organ Areas

At anthesis, two adjacent 50 cm row samples were collected to count the number of culms and spikes for each plot. A total of 20 stems with spikes were randomly selected from the sample to measure and record the green organ areas. For the leaf lamina, an automatic planimeter (LI-3000 area meter, LI-COR, Lincoln, NE) was used to measure the leaf area. Non-flat surfaces organs, spike length, peduncle length and stem length were measured with a ruler; spike thickness, spike width, the diameter of the peduncle and each node were measured at the middle of the organ using Vernier calipers. We measured the planar area rather than the total green area. The total surface area of the spike was calculating as a cylinder:

(1) $$Spike\;surface\;area=L\times \pi \times D+\pi \times {\left(\frac{D}{2}\right)}^{2}$$

where *L* is length from the rachis to the top of the spike, excluding the length of the awns, and *D* is the average spike thickness and width. We did not use this method to measure the area of the awn directly, and since the area of the awns is proportional to the spike length and width, the spike surface area (excluding the awns) is overestimated, compensating for the underestimation due to neglecting the awns.

The spike area (effective surface area) was calculated as the spike surface area multiplied by 0.5, *Spike area* = *Spike surface area* × 0.5, which is better correlated with light interception.

The total surface area of the stem with the leaf sheath was calculated as:

(2) Stem surface area=L1×π×D1+L2×π×D2

where *L*1 is the length of the peduncle, *D*1 is the diameter of thevpeduncle; *L*2 is the length of the stem and sheath excluding the length of the peduncle, and *D*2 is the average diameter of the culm and sheath. The stem area was calculated the same as the spike: *Stem area* = *Stem surface area ×* 0.5.

The *LAI* was calculated using only one side of the leaf tissue area per ground unit. The non-leaf area index (*NLAI*) was calculated as:

(3) $$NLAI=\frac{\left(Spike\;area+Stem\;area\right)\times Number\;of\;culms}{Land\;area}$$

The green area index (*GAI*) was calculated as:

(4) GAI=LAI+NLAI

#### 4.2.2. Light Interception

The photosynthetically active radiation (PAR) intercepted by the structures in different layers was calculated under different irrigations at anthesis in 2016/2017 and 2017/2018. For this, measurements were taken between 11:00 to 13:00 using a hand-held ceptometer (Model CI-150, CID, Inc., Vancouver, WA, USA). The PAR was measured at defined levels within the crop canopy (i.e., just above the plant canopy, below the spike, below the flag leaf, below the penultimate leaf, at the soil level). The height of the selected spike, flag leaf, and penultimate leaf was based on the height of most plants in the plot. Light interception (*LI*) was calculated using the following equation:

(5) $$LI=(1-I_{b}/I_{a})\times 100\%$$

where *I_a_* is the PAR above the layer and *I_b_* is the PAR below the layer.

#### 4.2.3. The Canopy Temperature and Temperature of Wheat Organs

The CT and temperature of wheat organs (e.g., spike, flag leaf) were measured at anthesis, 10 days after anthesis (DAA) and 20 DAA using a handheld infrared thermometer (Sixth Sense LT 300, Instrument, South Burlington, VI, USA). For each plot,10 plants were selected to measure the temperature of the spike, flag leaf, center of the stem and base of the stem. The *CT* measurements were made at ground level along each of the plots from 0.5 m above the canopy, angled to avoid bare soil and directed specifically at the part of the plot most exposed to the sun. A sensor was installed at a height of 1.5 m in a nearby uncropped plot to collect air temperature data.

Canopy temperature depression (*CTD*) is expressed as the difference between the air temperature (*T_air_*) and canopy temperature:

(6) $$CTD=T_{air}-CT$$

#### 4.2.4. The Normalized Difference Vegetation Index

For each plot, the normalized difference vegetation index (*NDVI*) was measured using a handheld crop sensor (GreenSeeker RT100, NTech Industries, Ukiah, CA, USA) at 70–90 cm above the plant canopy every day after the booting stage. The *NDVI* was calculated using the following equation:

(7) $$NDVI=\frac{NIR-R}{NIR+R}$$

where *NIR* and *R* are the measured reflectance near the infrared and red spectral bands, respectively.

#### 4.2.5. Canopy Apparent Photosynthesis

The rates of *CAP* were measured using a custom-made chamber with an infrared gas analyzer (LI-6400, LI-COR, Lincoln, NE, USA). The chamber consisted of a 1 × 1 × 1 m aluminum frame covered with mylar film and two battery-powered 50 W fans. The chamber was tall enough for the wheat plants to be contained without affecting the canopy structure. Measurements with three replicates were taken between 9:00 and 11:30 at the booting stages, anthesis stages, 14 DAA, 24 DAA and 29 DAA. The diurnal changes in *CAP* were measured every two hours from 8:00 to 20:00 at 1 DAA. The decrease in carbon dioxide concentration was monitored within 5 min after the plants were enclosed in the chamber. After finishing the *CAP* measurement, the respiration rates (*Rd*) were measured by placing a black opaque cloth over the chamber. Soil respiration (*R_so_*_il_) was measured in a nearby plot under the same treatment with all the plants removed at ground level. The *CAP*, *Rd* and *R_soil_* (mg CO_2_ s^−1^ m^−2^) were calculated using the following equation:

(8) $$CAP\prime (or\;Rd\prime ,R_{soil})=\frac{(c_{0}-c_{1})\times V}{(t_{1}-t_{0})\times S}\times (\frac{44}{22.4}\times \frac{P}{101.3}\times \frac{273}{273+T})$$

(9) $$CAP(or\;Rd)=CAP\prime (or\;Rd\prime )+|R_{soil}|$$

where *c*_0_ and *c*_1_ are the initial and final concentrations of carbon dioxide (mg L^−1^); *t*_0_ and *t*_1_ are the start and end times (s); *V* is the volume of chamber (L); *S* is the ground area (m^2^); *P* is the air pressure (Pa) and *T* is the air temperature (°C).

#### 4.2.6. Grain Yield, Soil Water Content, Evapotranspiration (ET), and WUE

At physiological maturity, spikes from an approximately 2 m^2^ area of each plot were harvested by hand, and samples were dried in an oven at 60–75 °C until a constant weight was obtained to investigate the yield.

Soil samples were taken from 0 to 200 cm in layer segments of 20 cm using a hand-held soil corer at the sowing stage, anthesis and maturity. The soil samples were dried in an oven at 105 °C for 48 h, then weighed, and the weight was recorded. The soil gravimetric water content (%; GWC) was measured as the water content (g) divided by the oven-dried weight (g). The soil volumetric water content (%; VWC) was calculated as the GWC multiplied by the soil bulk density. The soil water content (mm) was calculated as the VWC multiplied by the soil thickness (mm). The capillary rise and water drainage were negligible, and no surface runoff occurred from the experimental plots. The *ET* under varying irrigation regimes was calculated using the water balance equation:

(10) ET=SWD+P+I

where *ET* is evapotranspiration (mm), *SWD* is the variation in moisture from sowing and maturity (mm), *P* is precipitation (mm) and *I* is irrigation amount (mm).

The WUE (kg m^−3^) values were defined as the ratio of the grain yield to the crop evapotranspiration (*ET*).

### 4.3. Statistical Analysis

Analyses of variances (ANOVAs) were performed using SPSS version 20.0 (IBM, Armonk, NY, USA) [46]. The least significant difference (LSD) test was used to evaluate differences among treatments, and the significance level was set to 0.05 or 0.01 confidence levels. The data were plotted using Origin Pro 2019 (Origin Lab Corporation, Northampton, MA, USA) [47], R version 4.1.1 [48] and Adobe Illustrator CC version 21.0.0 [49].

## 5. Conclusions

Increasing irrigation times improved the wheat yield, but the W_2_ treatment had a comparable yield with the W_3_ treatment and a higher WUE. The CAPs under the W_3_ treatment were consistently lower than in the W_1_/W_2_ treatments during the post-anthesis period, suggesting a lower effective utilization of light energy under the W_3_ treatment. In addition, there were no significant differences in SWC at 0–200 cm between the W_1_/W_2_ and W_3_ treatments at the anthesis stage. This indicates that the lower effective utilization of light energy under the W_3_ treatment is caused by a change in canopy structure, not soil moisture. Increasing irrigation times improved the LAI and NLAI at anthesis and increased the LI of the spike and canopy, but decreased the LI of the lower strata leaves and accelerated the rate of leaf senescence, which results in a decreased CAP under the W_3_ treatment. As there is no clear correlation between the NDVI, other canopy photosynthetic parameters and canopy architecture indicators, it cannot reflect vertical changes in canopy structure. Significant correlations were found in this study between the CT and yield, while the organ temperature of the center and base of the stem were closely positively related with the LI of the lower layers. Overall, a reasonable canopy architecture played an important role in improving the photosynthetic potential later during grain filling. The W_2_ treatment was the best irrigation scheme for saving water and obtaining a comparable yield and a higher WUE of winter wheat in the NCP. The CT and organ temperature can be important parameters used to reflect the canopy architecture.

## Figures and Tables

**Table 1:** The area and ratio of green photosynthetic organs per stem at anthesis in 2015/2019.

Year	Irrigation Times	Leaf Area and Ratio (cm^2^, %)	Stem Area and Ratio (cm^2^, %)	Spike Area and Ratio (cm^2^, %)	Total Area (cm^2^)
2015/2016	W_0_	46.2 ± 0.5 ^b^(48.7)	38.4 ± 1.1 ^a^(40.4)	10.4 ± 0.1 ^b^(10.9)	95.0 ± 1.5 ^b^
	W_1_	56.6 ± 3.5 ^a^(50.4)	43.9 ± 1.7 ^a^(39.2)	11.6 ± 0.3 ^a^(10.4)	112.1 ± 5.5 ^a^
	W_3_	-	-	-	-
2016/2017	W_0_	33.5 ± 1.3 ^b^(40.2)	39.3 ± 0.8 ^b^(47.3)	10.4 ± 0.2 ^b^(12.5)	83.3 ± 1.4 ^b^
	W_1_	45.5 ± 1.9 ^a^(45.2)	44.0 ± 1.1 ^a^(44.0)	10.8 ± 0.4 ^ab^(10.8)	100.3 ± 2.9 ^a^
	W_3_	49.1 ± 0.7 ^a^(49.2)	39.1 ± 1.0 ^b^(39.1)	11.8 ± 0.4 ^a^(11.8)	100.0 ± 1.1 ^a^
2017/2018	W_0_	37.7 ± 1.5 ^b^(52.2)	24.8 ± 0.5 ^c^(34.3)	9.7 ± 0.2 ^b^(13.4)	72.1 ± 2.0 ^b^
	W_1_	38.4 ± 3.2 ^b^(51.1)	26.8 ± 0.7 ^b^(35.9)	9.7 ± 0.2 ^b^(13.0)	74.8 ± 3.9 ^b^
	W_3_	61.5 ± 3.0 ^a^(58.1)	32.6 ± 0.7 ^a^(30.8)	11.8 ± 0.3 ^a^(11.2)	105.9 ± 3.9 ^a^
2018/2019	W_0_	45.7 ± 2.9 ^b^(51.0)	34.7 ± 1.6 ^b^(38.8)	9.2 ± 0.3 ^b^(10.3)	89.6 ± 4.4 ^b^
	W_1_	53.6 ± 7.5 ^b^(50.4)	41.5 ± 4.0 ^b^(39.4)	10.6 ± 0.2 ^a^(10.2)	105.7 ± 11.6 ^b^
	W_3_	77.3 ± 7.0 ^a^(54.0)	54.1 ± 3.5 ^a^(37.9)	11.4 ± 0.5 ^a^(8.1)	142.8 ± 10.7 ^a^

Each value represents the mean ± SE. Values in parentheses indicate the ratio of the green photosynthetic organs. Different lowercase letters indicate significant differences based on an LSD test (*p* < 0.05) among irrigation treatments.

**Table 2:** Canopy green organs’ area index with different irrigation treatment at anthesis in 2015/2019.

Year	Irrigation Times	LAI	NLAI	GAI
2015/2016	W_0_	3.83 ± 0.10 ^b^	4.05 ± 0.16 ^b^	7.88 ± 0.25 ^b^
	W_1_	5.30 ± 0.30 ^a^	5.20 ± 0.17 ^a^	10.50 ± 0.47 ^a^
	W_3_	-	-	-
2016/2017	W_0_	2.74 ± 0.11 ^c^	4.06 ± 0.08 ^b^	6.80 ± 0.12 ^c^
	W_1_	3.98 ± 0.17 ^b^	4.80 ± 0.11 ^a^	8.78 ± 0.26 ^b^
	W_3_	4.88 ± 0.06 ^a^	5.06 ± 0.13 ^a^	9.94 ± 0.11 ^a^
2017/2018	W_0_	3.28 ± 0.05 ^c^	3.01 ± 0.12 ^b^	6.29 ± 0.17 ^c^
	W_1_	4.22 ± 0.21 ^b^	4.02 ± 0.07 ^a^	8.24 ± 0.15 ^b^
	W_3_	5.70 ± 0.27 ^a^	4.11 ± 0.08 ^a^	9.81 ± 0.33 ^a^
2018/2019	W_0_	3.70 ± 0.24 ^b^	3.55 ± 0.15 ^b^	7.25 ± 0.36 ^b^
	W_1_	4.42 ± 0.62 ^ab^	4.29 ± 0.34 ^ab^	8.72 ± 0.95 ^ab^
	W_3_	5.39 ± 0.49 ^a^	4.57 ± 0.26 ^a^	9.96 ± 0.74 ^a^

Note: leaf area index (LAI), non-leaf area index (NLAI) and green area index (GAI). Each value represents the mean ± SE. Different lowercase letters indicate significant differences based on an LSD test (*p* < 0.05) among irrigation treatments.

**Table 3:** Canopy temperature and canopy temperature depression at anthesis and the grain filling stage in 2016/2018.

Year	Irrigation Times	CT (°C)			CTD (°C)		
		1 DAA	10 DAA	20 DAA	1DAA	10 DAA	20 DAA
2016/2017	W_0_	25.17 ^a^	28.78 ^a^	33.17 ^a^	4.23 ^c^	4.02 ^d^	3.24 ^d^
	W_1_	24.95 ^b^	28.13 ^b^	32.76 ^b^	4.45 ^b^	4.67 ^c^	3.64 ^c^
	W_2_	-	27.84 ^c^	32.32 ^c^	-	4.96 ^b^	4.08 ^b^
	W_3_	24.68 ^c^	27.43 ^d^	32.07 ^d^	4.72 ^a^	5.37 ^a^	4.33 ^a^
2017/2018	W_0_	24.82 ^a^	19.56 ^a^	29.44 ^a^	3.88 ^c^	4.07 ^c^	3.56 ^c^
	W_1_	24.17 ^b^	19.12 ^b^	28.70 ^b^	4.53 ^b^	4.39 ^b^	4.30 ^b^
	W_2_	-	18.27 ^c^	28.67 ^b^	-	4.61 ^b^	4.33 ^b^
	W_3_	23.86 ^c^	17.73 ^d^	28.10 ^c^	4.84 ^a^	5.04 ^a^	4.90 ^a^

Note: Canopy temperature (CT), canopy temperature depression (CTD), days after anthesis (DAA). Each value represents the mean ± SE. Different lowercase letters indicate significant differences based on an LSD test (*p* < 0.05) among the irrigation treatments.

**Table 4:** Organ temperature at anthesis and grain filling stage in 2017/2018.

Time	Irrigation Times	Organ Temperature (°C)			
		Spike	Flag Leaf	Central of the Stem	Base of the Stem
1 DAA	W_0_	26.35 ^a^	23.19 ^a^	22.64 ^a^	23.91 ^a^
	W_1_	25.43 ^b^	22.72 ^b^	22.29 ^b^	23.77 ^b^
	W_2_	-	-	-	-
	W_3_	24.83 ^c^	21.86 ^c^	21.41 ^c^	22.92 ^c^
10 DAA	W_0_	20.63 ^a^	18.95 ^a^	18.91 ^a^	19.62 ^a^
	W_1_	19.54 ^b^	18.73 ^b^	18.48 ^b^	19.02 ^b^
	W_2_	19.04 ^c^	18.19 ^c^	18.16 ^c^	18.85 ^b^
	W_3_	18.51 ^d^	18.08 ^c^	17.96 ^d^	18.34 ^c^
20 DAA	W_0_	30.92 ^a^	27.87 ^a^	28.77 ^a^	30.83 ^a^
	W_1_	28.91 ^b^	27.12 ^b^	28.14 ^b^	30.28 ^ab^
	W_2_	28.33 ^c^	27.08 ^b^	27.91 ^b^	29.99 ^bc^
	W_3_	28.38 ^d^	26.88 ^c^	27.69 ^b^	29.51 ^c^

Note: Each value represents the mean value. Different lowercase letters indicate significant differences based on an LSD test (*p* < 0.05) among the irrigation treatments.

**Table 5:** Grain yield, ET and WUE of winter wheat under different treatments from 2015 to 2019.

Year	Irrigation Times	Yield (kg ha^−1^)	ET (mm)	WUE (kg m^−3^)
2015/2016	W_0_	6298 ± 63 ^c^	396.0 ± 13.1 ^c^	1.59 ± 0.02 ^b^
	W_1_	7702 ± 226 ^b^	444.9 ± 2.1 ^b^	1.73 ± 0.05 ^a^
	W_2_	8500 ± 30 ^a^	482.2 ± 4.1 ^a^	1.76 ± 0.01 ^a^
	W_3_	-	-	-
2016/2017	W_0_	6370 ± 101 ^c^	392.6 ± 12.7 ^c^	1.63 ± 0.05 ^b^
	W_1_	7084 ± 191 ^b^	404.3 ± 3.4 ^bc^	1.75 ± 0.01 ^ab^
	W_2_	7736 ± 91 ^a^	430.1 ± 1.6 ^b^	1.80 ± 0.01 ^a^
	W_3_	8059 ± 158 ^a^	464.0 ± 15.4 ^a^	1.74 ± 0.06 ^ab^
2017/2018	W_0_	6415 ± 198 ^c^	354.8 ± 9.3 ^c^	1.81 ± 0.06 ^bc^
	W_1_	7519 ± 104 ^b^	391.0 ± 13.7 ^b^	1.92 ± 0.03 ^ab^
	W_2_	8135 ± 50 ^a^	417.2 ± 9.2 ^b^	1.95 ± 0.01 ^a^
	W_3_	8291 ± 194 ^a^	488.1 ± 2.5 ^a^	1.70 ± 0.04 ^c^
2018/2019	W_0_	5446 ± 118 ^d^	334.2 ± 11.1 ^c^	1.63 ± 0.04 ^b^
	W_1_	6247 ± 105 ^c^	352.5 ± 9.0 ^c^	1.77 ± 0.03 ^a^
	W_2_	7264 ± 89 ^b^	422.5 ± 2.7 ^b^	1.72 ± 0.02 ^ab^
	W_3_	7883 ± 240 ^a^	469.8 ± 2.4 ^a^	1.68 ± 0.05 ^ab^

Note: each value represents the mean ± SE. Different lowercase letters indicate significant differences based on an LSD test (*p* < 0.05) among the irrigation treatments.

**Table 6:** Details of irrigation treatment in the present study.

Irrigation Treatment	Irrigation Amount (mm)			
	Before Sowing Stage	Tillering Stage	Jointing Stage	Anthesis Stage ^2^
W_0_	100 mm	-	-	-
W_1_	100 mm	-	75 mm	-
W_2_	100 mm	-	75 mm	75 mm
W_3_ ^1^	100 mm	75 mm	75 mm	75 mm

^1^ The W_3_ treatment was only applied in 2016/2017, 2017/2018 and 2018/2019. ^2^ The anthesis dates were 3 May 2016, 8 May 2017, 9 May 2018 and 8 May 2019.

## Data Availability

The data presented in this study are available from the corresponding author, Yanmei Gao, upon reasonable request.
